# Factors influencing spontaneous hypothermia after emergency trauma and the construction of a predictive model

**DOI:** 10.1515/biol-2022-0862

**Published:** 2024-04-20

**Authors:** Xia Feng, Fangxiang Zhu, Anhua Qiao, Wenfang Li, Ying Jiang, Zengtao Han, Lan Dong

**Affiliations:** Emergency Department, Shanghai Chang Zheng Hospital, Shanghai 200003, China; Nursing Department, Shanghai Chang Zheng Hospital, Shanghai 200003, China; Cerebrovascular Diseases Center, Department of Neurosurgery Renji Hospital, Shanghai 201112, China

**Keywords:** emergency trauma, spontaneous hypothermia, influencing factors, predictive model

## Abstract

This study aimed to investigate spontaneous hypothermia among emergency trauma patients and develop a predictive model. A cohort of 162 emergency trauma patients was categorized into hypothermic (*n* = 61) and control (*n* = 101) groups, with trauma severity assessed using the modified Glasgow Coma Scale (GCS). Univariate analysis revealed significant differences between the groups in trauma severity, posture, garment wetness, warming measures, pre-hospital fluid resuscitation, and modified GCS scores (*P* < 0.05). The hypothermic group exhibited lower prothrombin time compared to the control group (*P* < 0.05). A logistic regression model was constructed, expressed as *Y* = 25.76 − 1.030*X*_1_ + 0.725*X*_2_ + 0.922*X*_3_ − 0.750*X*_4_ − 0.57*X*_6_, and its fit was evaluated using the Hosmer–Lemeshow test. The receiver operating characteristic curve demonstrated an area under the curve of 0.871, with 81.2% sensitivity and 79.5% specificity. The Youden index identified the optimal predictive cut-off at its highest (0.58). Validation results included 86.21% sensitivity, 82.93% specificity, and 84.29% accuracy. Risk factors for spontaneous hypothermia after emergency trauma encompassed trauma severity, posture during consultation, clothing dampness upon admission, warming measures during transfer, pre-hospital fluid resuscitation, and modified GCS scores. The risk prediction model demonstrated high accuracy, enabling effective assessment of spontaneous hypothermia risk in emergency trauma patients and facilitating preventive measures.

## 1 Introduction

Spontaneous hypothermia after trauma is a common complication encountered during the trauma process, with an incidence rate ranging from 12 to 66% [1,2]. Its severity can be categorized into three classes: mild hypothermia (36–34°C), moderate hypothermia (34–32°C), and severe hypothermia (<32°C). The combination of hypothermia, acidosis, and coagulopathy is known as the “lethal triad” in trauma patients, and those who develop spontaneous hypothermia post-trauma have a poorer prognosis [3]. Emergency trauma patients frequently experience spontaneous hypothermia, a potentially fatal complication that can seriously affect the patient’s prognosis and life during resuscitation. Spontaneous hypothermia is defined as a core body temperature below 35°C induced by a variety of conditions, including heat loss, metabolic problems, medication usage, and environmental variables. Spontaneous hypothermia is a major concern for emergency trauma patients that requires adequate care and avoidance. Both domestic and international trauma care guidelines [4,5] emphasize the proactive prevention and treatment of hypothermia following injury. Currently, a significant portion of research conducted both domestically and abroad is centered on investigating the negative consequences associated with spontaneous hypothermia subsequent to trauma.

Nevertheless, the current body of literature regarding the occurrence and determinants of spontaneous hypothermia following acute trauma is very scarce, with a majority of the available studies being retrospective. There exists a conspicuous deficiency in the knowledge and prioritization of post-trauma spontaneous hypothermia among medical and emergency personnel in our nation. The precise occurrence and factors contributing to this phenomenon remain uncertain. Studies indicate that early identification and timely correction of potential patients can effectively improve the prognosis of trauma patients with spontaneous hypothermia, reducing their risk of mortality [6]. However, most existing research focuses on the adverse outcomes associated with spontaneous hypothermia, with limited reports on risk assessment of spontaneous hypothermia post-trauma. Currently, despite the advancements made in the prevention and management of spontaneous hypothermia in emergency trauma patients, several obstacles persist. First, our understanding of the etiology and pathogenesis of spontaneous hypothermia is not deep enough. Second, there are differences in current preventive measures and treatment methods and a lack of consistent guidance and consensus. Hence, it is imperative to undertake additional research endeavors in order to enhance our comprehension of the disease and develop more productive treatment strategies for patients. Therefore, the primary objective of this research is to investigate the underlying mechanism and identify the risk variables associated with spontaneous hypothermia in emergency trauma patients.

Additionally, the study aims to assess the efficacy of current preventive interventions and put forth recommendations for further enhancements. This study aims to establish a robust foundation for clinical practice and policy decision-making, with the ultimate goal of enhancing survival and recovery rates among emergency trauma patients. Moreover, we hope to investigate the components that influence spontaneous hypothermia in patients following emergency trauma and, based on our findings, develop a prediction model to predict its development, which will serve as a reference for clinical intervention.

## 2 Materials and methods

### 2.1 Study data

This study employed a cross-sectional study design, with acute trauma patients admitted to our hospital’s emergency department from January 2019 to January 2021. They were thoroughly screened in accordance with the established inclusion criteria, and a temperature of <36°C was selected as the diagnostic basis for hypothermia in trauma patients [7]. Inclusion criteria were age ≥18 years old and complete case data. Exclusion criteria included: suffering from serious diseases before trauma, such as malignant tumors, systemic infections, and severe malnutrition. Sample selection: the case record system and hospitalization database are used to identify patients who align with the inclusion criteria for the study. Data collection: the medical records of all patients included in the study were obtained, and study investigators collected the following data: basic demographic information, characteristics of the emergency trauma, factors contributing to the condition, assessment of its severity, drugs used during therapy, and vital signs recorded throughout hospitalization. The final study sample consisted of a total of 162 emergency trauma patients after excluding 38 subjects based on the predetermined exclusion criteria.


**Informed consent:** Informed consent has been obtained from all individuals included in this study.
**Ethical approval:** The research related to human use has been complied with all the relevant national regulations, institutional policies and in accordance with the tenets of the Helsinki Declaration, and has been approved by the authors’ institutional review board or equivalent committee.

### 2.2 Methods

#### 2.2.1 Trauma assessment

The modified Glasgow Coma Scale (GCS) [8] was used to assess the severity of patient trauma. This encompassed GCS ratings (verbal, eye response, and motor function), respiration, and systolic blood pressure. Scores ranged from 3 to 15, with a lower score indicating heightened trauma severity. Based on the combined scores, trauma patients were categorized into three levels: mild trauma (12–15 points), moderate trauma (7–11 points), and severe trauma (≤6 points).

#### 2.2.2 Data collection

Following a comprehensive examination of relevant literature and the integration of valuable perspectives from medical experts, a questionnaire was developed. It included demographic information such as age; gender; body mass index (BMI); educational level; trauma type, including car accidents, falls from heights, and stab wounds; on-site status: patient’s position at the time of consultation (squatting, lying down, standing, or trapped); measures taken for warming and fluid administration during transit; time taken to reach the emergency room; and post-admission assessments like vital signs, trauma evaluation, and room temperature.

#### 2.2.3 Laboratory examination

The study involved the observation of various coagulation function indicators in patients, including thrombin time (TT), activated partial thromboplastin time (APTT), prothrombin time (PT), antithrombin III (AT-III), fibrinogen (FIB), and D-dimer.

#### 2.2.4 Temperature monitoring

The patient’s body temperature was monitored using a calibrated infrared tympanic thermometer. In situations where the tympanic thermometer is deemed inappropriate, other thermometers like forehead or mercury thermometers are employed.

### 2.3 Statistical analysis

Collected experimental data were analyzed using SPSS 21.0. After passing the quantitative data, which passed the Kolmogorov–Smirnov test for normality, it was represented by *X* ± S. Independent sample *t*-tests were used for comparison between the two groups, while count data were represented as numbers or rates, with *χ* ^2^ tests used for intergroup comparison. Variables with significant differences were subjected to multivariate logistic regression analysis, with the Hosmer–Lemeshow test validating the model’s fit. A smaller *P*-value indicates a poorer fit. Receiver operating characteristic (ROC) curve was plotted to evaluate the applicability of the hypothermia prediction model. A *P*-value <0.05 was considered statistically significant.

## 3 Results

### 3.1 General information of the patients

**Figure 1:**
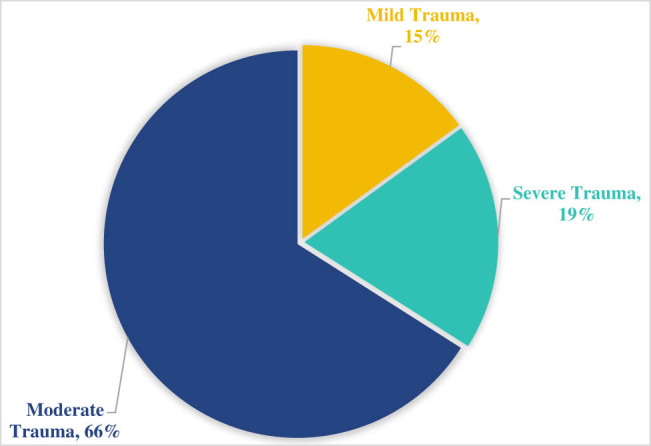
Composition of injury types.

There were 162 patients included in the study, of which 83 were male and 79 were female. The age range was 20–76 years, with an average of 44.54 ± 11.15 years. Types of injuries included 99 cases of car accidents, 29 cases of high fall injuries, 9 cases of stab wounds, and 25 other cases (Figure 1).

**Figure 2:**
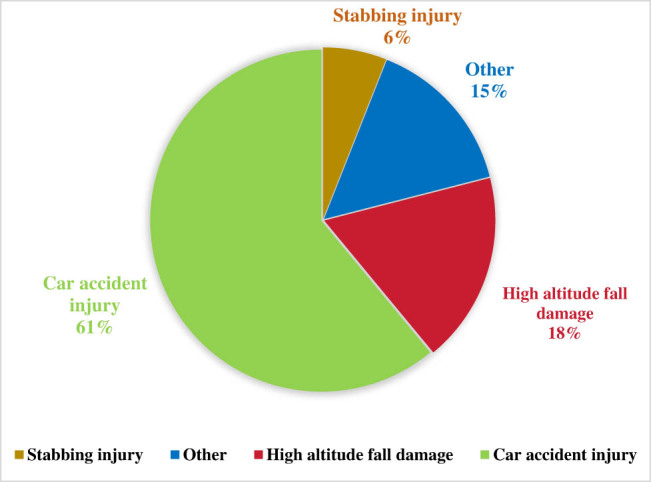
Distribution of trauma severity.

Figure 2 displays the distribution of trauma cases, with 25 cases classified as mild trauma, 107 cases categorized as moderate trauma, and 30 cases designated as severe trauma. Out of the entire cohort, a group of 61 patients exhibited spontaneous hypothermia and were classified as the hypothermic group, whereas a control group consisting of 101 patients without hypothermia was also included.

### 3.2 Univariate analysis of spontaneous hypothermia after emergency trauma

There were significant discrepancies observed in various factors between the two groups, including the extent of trauma, body position upon admission, wetness of clothing upon admission, warming measures during transfer, pre-hospital fluid administration, and modified GCS score (*t* = 23.402, 12.113, 4.659, 16.951, 7.778, 10.970; *P* = 0.000, 0.007, 0.031, 0.000, 0.005, and 0.000, respectively). However, no statistical differences were observed in age, gender, BMI, educational level, type of injury, time taken to reach the emergency room, blood pressure, heart rate, respiration, and emergency room temperature between the two groups. Detailed results are presented in Table 1.

**Table 1:** Univariate analysis of spontaneous hypothermia after emergency trauma

Variable	The hypothermic group (*n* = 61)	The control group (*n* = 101)	*t*/*χ* ^2^	*P*
Age (years)	45.87 ± 10.26	43.89 ± 10.76	1.155	0.250
**Gender**				
Male	34	48	1.026	0.311
Female	27	53		
BMI (kg/m^2^)	23.48 ± 1.27	23.09 ± 1.36	1.812	0.072
**Educational level**				
High school or below	37	65	0.223	0.637
Bachelor’s degree or below	24	36		
**Trauma severity**				
Mild	5	22	23.402	<0.001
Moderate	33	71		
Severe	23	8		
**Type of injury**				
Car accident injury	34	69	2.599	0.107
Non-car accident injury	27	32		
**Body position at admission**				
Crouching	10	5	12.113	0.007
Lying flat	37	85		
Standing	8	5		
Trapped	6	6		
**Wetness of clothing upon admission**				
Yes	31	34	4.659	0.031
No	30	67		
**Warming measures during transfer**				
Yes	10	49	16.951	<0.001
No	51	52		
**Pre-hospital fluid administration**				
Yes	4	23	7.778	0.005
No	57	74		
**Time taken to reach the emergency room**				
<30 min	41	73	0.468	0.494
≥30 min	20	28		
Systolic blood pressure (mmHg)	125.95 ± 15.27	128.61 ± 16.18	1.035	0.302
Diastolic blood pressure (mmHg)	75.98 ± 11.76	77.23 ± 12.03	0.646	0.519
Heart rate (beats/min)	85.75 ± 15.43	86.43 ± 15.25	0.274	0.785
Respiration rate (breaths/min)	20.68 ± 3.47	19.84 ± 3.52	1.479	0.141
Emergency room temperature (°C)	23.57 ± 1.68	24.05 ± 1.72	1.736	0.084
The modified GCS score	9.38 ± 1.19	11.82 ± 1.47	10.970	<0.001

### 3.3 Coagulation function in both patient groups

Compared to the control group, patients in the hypothermia group showed a significant decrease in PT (*t* = 3.146, *P* = 0.002). There was no statistically significant difference between the two groups regarding TT, APTT, AT-III, FIB, and D-dimer (Table 2).

**Table 2:** Coagulation function of both patient groups

Parameter	The hypothermic group (*n* = 61)	The control group (*n* = 101)	*t*	*P*
TT (s)	16.14 ± 0.72	16.33 ± 0.87	1.434	0.153
APTT (s)	26.81 ± 2.89	27.54 ± 2.68	1.631	0.105
PT (s)	7.33 ± 0.72	7.74 ± 0.85	3.146	0.002
AT-Ⅲ (%)	97.29 ± 3.32	96.68 ± 2.57	1.309	0.192
FIB (g/L)	6.05 ± 0.62	5.87 ± 0.53	1.963	0.051
D-dimer (mg/L)	0.57 ± 0.12	0.54 ± 0.13	1.464	0.145

### 3.4 Establishment of the spontaneous hypothermia prediction model

A logistic regression analysis was performed, with the occurrence of post-emergency trauma hypothermia in patients as the dependent variable, classified as “Yes” (1) or “No” (0). Independent variables were selected based on substantial differences observed in univariate comparisons (Table 3). The results revealed that trauma severity (*X*_1_), body position at consultation (*X*_2_), wet clothing upon admission (*X*_3_), warming measures taken during transfer (*X*_4_), pre-hospital fluid administration (*X*_5_), and the modified GCS score (*X*_6_) were correlated with the occurrence of post-emergency trauma spontaneous hypothermia. The predictive model is given by *Y* = 25.76 − 1.030*X*_1_ + 0.725*X*_2_ + 0.922*X*_3_ − 0.750*X*_4_ − 0.57*X*_6_ (Table 4).

**Table 3:** Assignment of independent variables

Independent variable	Assignment
Trauma severity (*X*_1_)	Mild = 1, moderate = 2, severe = 3
Body position at consultation (*X*_2_)	Lying flat = 1, standing = 2, trapped = 3, crouching = 4
Wet clothing upon admission (*X*_3_)	Yes = 1, No = 2
Warming measures taken during transfer (*X*_4_)	Yes = 1, No = 2
Pre-hospital fluid administration (*X*_5_)	Yes = 1, No = 2
The modified GCS score (*X*_6_)	≤6 points = 1, 7–11 points = 2, 12–15 points = 3

**Table 4:** Results of the logistic regression analysis

Variable	*β*	SE	Ward	*P*	OR	95% CI
Trauma severity (*X*_1_)	−1.030	0.348	8.760	0.002	0.357	0.180–0.706
Body position at consultation (*X*_2_)	0.725	0.287	6.383	<0.001	2.065	1.176–3.624
Wet clothing on admission (*X*_3_)	0.922	0.332	7.723	<0.001	2.516	1.312–4.822
Warming measures during transport (*X*_4_)	−0.750	0.339	4.904	0.008	0.472	0.242–0.917
Pre-hospital fluid resuscitation (*X*_5_)	−0.709	0.324	4.792	<0.001	0.492	0.260–0.928
The modified GCS score (*X*_6_)	−0.556	0.247	5.082	0.013	0.573	0.353–0.929
Constant	25.76	5.64	10.376	0.022	—	—

### 3.5 Prediction model fitting and prediction performance

**Figure 3:**
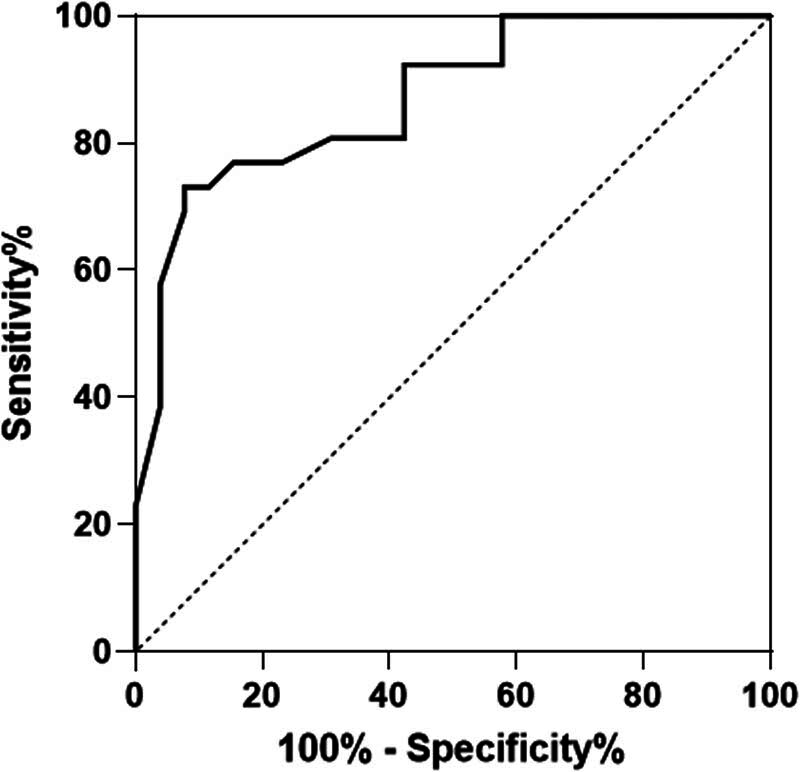
ROC curve for predicting spontaneous hypothermia after emergency trauma.

The Hosmer–Lemeshow test was used to validate the prediction model’s fit, and the resultant *P*-value was 0.125. The ROC curve for the model’s prediction of spontaneous hypothermia was plotted to assess its sensitivity and specificity further. The area under the ROC curve was determined to be 0.871, with a sensitivity of 81.2% and a specificity of 79.5% when the Youden Index’s highest value (0.58) was used as the prediction model’s threshold (Figure 3).

### 3.6 Validation of the spontaneous hypothermia risk prediction model

In the year 2022, a sample of 70 emergency trauma patients was selected randomly in order to assess and validate the effectiveness of the model’s application. A total of 29 instances of spontaneous hypothermia were observed, while the predictive model accurately identified this condition in 25 cases. However, there were four cases in which the model made incorrect predictions. Consequently, the sensitivity of the model, which measures its ability to identify cases of spontaneous hypothermia correctly, was determined to be 86.21%. In instances where spontaneous hypothermia did not occur (41 cases), the model predicted 34 cases, misjudging 7 cases, with a specificity of 82.93%. The overall accuracy rate of the model prediction was (25 + 34)/70 = 84.29% (Table 5).

**Table 5:** Validation of spontaneous hypothermia risk prediction model (*n*, %)

Method	Number of cases	Spontaneous hypothermia	No hypothermia
Model prediction	70	25 (35.71)	34 (48.57)
Actual diagnosis	70	29 (41.43)	41 (58.57)

## 4 Discussion

The occurrence of spontaneous hypothermia in emergency patients has the potential to inhibit coagulation-related enzyme-linked processes, hence impacting platelet function. Acidosis is correlated with reduced body temperature and blood loss due to its potential to induce vasoconstriction, hence contributing to metabolic dysfunction and facilitating the buildup of lactic acid [9]. Therefore, the rapid screening and timely correction of hypothermia are crucial. Nevertheless, as a result of the intricate nature of trauma situations and the unpredictability of trauma locations, precisely and promptly determining the actual body temperature of emergency trauma patients poses a significant challenge. The current instruments utilized for evaluating the risk of hypothermia demonstrate a lack of specificity. Consequently, there is a necessity to develop a predictive model for the occurrence of spontaneous hypothermia following a traumatic event.

In this study, the incidence rate of spontaneous hypothermia in the patients used for modeling was 37.65%, which is consistent with related literature [10–12]. Most of these were victims of car accidents, with a majority presenting with moderate injuries. Factors influencing the onset of hypothermia post-trauma include sociodemographic, environmental factors, pre-hospital emergency care, the trauma itself, and iatrogenic factors [13,14]. Univariate analysis in this study revealed that the severity of trauma, the patient’s position during consultation, damp clothing upon admission, warming measures taken during transfer, pre-hospital fluid administration, and the modified GCS score are significant factors influencing the onset of spontaneous hypothermia.

The more severe the trauma, the more the blood loss, leading to reductions in peripheral blood volume and hemoglobin concentration. This exacerbates cerebral tissue hypoxia and ischemia, affecting the central nervous system’s ability to regulate core temperature, leading to hypothermia. The modified GCS score also decreases accordingly [15,16]. The use of a supine position during the consultation has been observed as showing a higher likelihood of being associated with hypothermia, potentially attributable to the lower temperature of the ground surface. This particular posture enhances the surface area of contact between the patient and the ground, hence facilitating the process of heat loss.

Moreover, emergency patients in the supine position often have varying levels of consciousness impairment; their thermoregulation center might be inhibited by trauma, suppressing heat production [17,18]. The use of crucial warming interventions during the process of transfer, such as the utilization of blankets or quilts for insulation or the administration of warming fluids, can effectively mitigate the risk of hypothermia. Following an accident, the body’s reactive reaction may exhibit a reduction, particularly in cases of severe injuries where skin damage might compromise insulation and expedite heat loss. Therefore, early warmth, moisture isolation, and prevention of heat loss are essential to maintain the patient’s temperature [19,20].

The multivariate logistic regression analysis conducted for this study revealed the following factors as predictors of the risk of post-trauma spontaneous hypothermia: trauma severity (*X*_1_), body position at consultation (*X*_2_), wet clothing upon admission (*X*_3_), pre-hospital fluid administration (*X*_5_), and the modified GCS score (*X*_6_). The predictive model is *Y* = 25.76 − 1.030*X*_1_ + 0.725*X*_2_ + 0.922*X*_3_ − 0.750*X*_4_ − 0.57*X*_6_ and has been validated with satisfactory fitting effects. One of the factors to consider is the level of trauma experienced (*X*_1_): a substantial association is present between the severity of trauma and the incidence of spontaneous hypothermia among individuals receiving emergency trauma care. Severe trauma has the potential to induce enhanced heat loss and metabolic irregularities inside the body, consequently impacting the functionality of the thermoregulatory system. Furthermore, trauma can cause inflammatory reactions and the production of stress hormones, which can further disrupt body temperature control and increase the risk of spontaneous hypothermia. Position during medical treatment (*X*_2_): position is significant in the development of spontaneous hypothermia in emergency trauma patients. Hypothermia can occur in patients who are positioned in supine or other constrained postures due to limitations imposed by the surrounding environment, which can impede air circulation and result in heat loss.

Furthermore, it should be noted that specific body positions can also give rise to complications such as inadequate blood flow, muscular constriction, and nerve compression, impeding the regular processes of heat production and control. Wet clothing upon admission (*X*_3_): wet clothing can increase heat conduction and accelerate body temperature loss. Exposure of patients to a humid environment during injury or transportation can lead to a more rapid decline in body temperature due to the presence of moist clothing. Moreover, it is worth noting that surroundings with high humidity have the potential to readily induce infections and inflammatory responses, thus exacerbating the disruption of body temperature regulation. Taking warm measures during transportation (*X*_4_): emergency trauma patients may significantly reduce the likelihood of developing spontaneous hypothermia by implementing suitable warm-up protocols. Heating measures may consist of the provision of insulated sheets, the use of heating apparatus, and the lending of appropriate attire. These measures contribute to the elevation of the patient’s body temperature and the production of heat. Pre-hospital infusion (*X*_5_): pre-hospital infusion also has a significant impact on the occurrence of spontaneous hypothermia in emergency trauma patients. Blood volume may increase as a result of excessive infusion, thereby augmenting the area for heat dissipation and heat loss.

Furthermore, low-temperature liquid infusion during the infusion procedure may result in an additional decrease in body temperature for the patient. Revised GCS score (*X*_6_): patients who have experienced emergency trauma are frequently assessed for neurological function using the revised GCS. The body’s ability to perceive and regulate temperature may be impacted by neurological dysfunction, which is typically linked to lower corrected GCS scores. Consequently, among emergency trauma patients, a lower modified GCS score may be associated with a higher likelihood of spontaneous hypothermia. Although there are limited related reports, this model can aid in identifying trauma patients at risk for hypothermia and implementing targeted preventive measures, such as focusing on patients with moderate-to-severe trauma or those in prone positions and actively warming them during transfers. This model can help identify high-risk patients in order to take timely preventive measures and improve patient care. It can be integrated into the workflow of the emergency room to improve the efficiency of patient monitoring and care. However, this model may have limitations, such as limited predictive ability for events within a short time window. Meanwhile, potential directions for improving the model are proposed, such as adding more relevant variables or optimizing algorithms to improve accuracy and practicality. The prediction results based on this model can revise the pre admission and upon admission patient care guidelines of hospitals, and promote their promotion and application in emergency rooms and other medical institutions. More relevant research should be conducted in the future to improve the model and expand its application scope. For example, exploring its applicability in different populations or environments, verifying its accuracy and effectiveness, etc.

## 5 Conclusion

In conclusion, the current investigation performed univariate and multivariate logistic regression analysis to identify six primary risk factors associated with spontaneous hypothermia in emergency trauma patients. These risk factors include trauma severity, body position during treatment, wet clothing upon admission, measures implemented to preserve warmth during transportation, pre-hospital infusion, and correction of GCS scores. Additionally, this study developed hypothermia risk prediction models based on these identified risk factors. The model has been empirically validated to possess robust prediction capabilities and substantial therapeutic value. The present study has successfully validated a hypothermia risk prediction model and has shown its commendable predictive capability. This model can be of substantial use to clinical practitioners in evaluating the risk and devising preventive strategies for emergency trauma patients. The prompt discusses the need to promptly identify patients who are at high risk for adverse outcomes and implement appropriate measures.
